# Validation of a 3D Markerless Motion Capture Tool Using Multiple Pose and Depth Estimations for Quantitative Gait Analysis

**DOI:** 10.3390/s24227105

**Published:** 2024-11-05

**Authors:** Mathis D’Haene, Frédéric Chorin, Serge S. Colson, Olivier Guérin, Raphaël Zory, Elodie Piche

**Affiliations:** 1 Arts et Métiers—Institut de Biomécanique Humaine Georges Charpak, 75013 Paris, France; mathis.d_haene@ensam.eu; 2 Université Côte d’Azur, CHU, France; chorin.f@chu-nice.fr (F.C.); guerin.o@chu-nice.fr (O.G.); raphael.zory@univ-cotedazur.fr (R.Z.); piche.e@chu-nice.fr (E.P.); 3 Université Côte d’Azur, LAMHESS, France; 4 Université Côte d’Azur, CNRS, INSERM, IRCAN, France; 5 Institut Universitaire de France (IUF), 75005 Paris, France

**Keywords:** 3D markerless motion capture, quantitative gait analysis, pose estimation, stereoscopic cameras, depth estimation

## Abstract

Gait analysis is essential for evaluating walking patterns and identifying functional limitations. Traditional marker-based motion capture tools are costly, time-consuming, and require skilled operators. This study evaluated a 3D Marker-less Motion Capture (3D MMC) system using pose and depth estimations with the gold-standard Motion Capture (MOCAP) system for measuring hip and knee joint angles during gait at three speeds (0.7, 1.0, 1.3 m/s). Fifteen healthy participants performed gait tasks which were captured by both systems. The 3D MMC system demonstrated good accuracy (LCC > 0.96) and excellent inter-session reliability (RMSE < 3°). However, moderate-to-high accuracy with constant biases was observed during specific gait events, due to differences in sample rates and kinematic methods. Limitations include the use of only healthy participants and limited key points in the pose estimation model. The 3D MMC system shows potential as a reliable tool for gait analysis, offering enhanced usability for clinical and research applications.

## 1. Introduction

Gait analysis is a vital tool for clinicians to evaluate walking patterns and to detect functional limitations. It is often added into traditional physical tests such as the 6 min walk test (6MWT) and the Timed-Up-and-Go (TUG) test [1,2]. In general, these tests are assessed qualitatively by the clinicians and self-reported by the patient [3,4], and some quantitative values such as gait speed or distance covered might also be provided by the clinician. However, these evaluations suffer from inter-observer variability and human error, reducing their precision and clinical relevance. Moreover, a single visual assessment does not capture all of the desired quantitative values at once, potentially overlooking interesting features that could enable a broader and more precise analysis [5,6]. Today, to obtain a precise evaluation of the gait, 3D Motion Capture (MOCAP) is used. Combining infrared cameras, reflective sensors and force plates, this technology is considered the gold standard of quantitative gait analysis. However, its limitations make its deployment in clinics difficult. Indeed, 3D motion capture is expensive, requires operator proficiency, and is time-consuming, which slows down its usage. Furthermore, the accuracy of lower limb kinematics is influenced by correct marker placement and soft tissue artifacts [7,8,9,10].

Over the last decade, advancements in artificial intelligence have led to the development of Markerless Motion Capture (MMC). MMC enables human pose estimation from digital camera footages [11]. Being cost and time effective, this technology, which uses open-source pose estimators [12], could be a valuable clinical tool, allowing clinicians to easily evaluate a patient’s walking patterns. While 2D pose estimation (2D MMC) has shown promising results [13,14,15], 3D pose estimation techniques (3D MMC) remain relatively new. Recent studies utilizing RGB cameras and triangulation methods during treadmill or over-ground activities have shown promise [16,17,18]. Similarly, RGB-D cameras like commercial Microsoft Kinect have been employed to combine depth and 2D pose estimations for gait and sit-to-stand assessments, demonstrating good agreement with traditional MOCAP [19,20,21,22].

However, using a single Kinect camera for motion analysis leads to body occlusions issues, and using multiple Kinect cameras simultaneously can introduce accuracy and stability challenges due to how the cameras detect body key points [23]. At the same time, a simple method combining a single depth camera with a separate pose estimation tool has shown promising results for identifying 3D body key points [24]. Nevertheless, this approach has not been yet tested with multiple depth cameras.

Thus, the aim of this study was to compare two motion capture systems for quantitative gait analysis in healthy adults: a simple 3D MMC system using a combination of multiple depth cameras and a pose estimator, against the OptiTrack optoelectronic MOCAP system (NaturalPoint, Inc., Corvallis, OR, USA), considered the gold standard. The primary hypothesis was that the markerless system would measure hip and knee joint angles in the sagittal plane with good accuracy compared to MOCAP. The secondary hypothesis proposed that the markerless system would demonstrate an excellent test–retest reliability. The findings show that the 3D MMC system exhibits excellent test–retest reliability and a moderate-to-good accuracy when compared to MOCAP, suggesting its potential as an accessible option for clinicians.

## 2. Materials and Methods

### 2.1. Participants

Fifteen young healthy participants (8 females, 7 males, age 27.8 years ± 4.5, height 175 cm ± 10, body mass 70 kg ± 12) were recruited. The exclusion criteria included musculoskeletal injuries and the inability to walk without assistance.

### 2.2. Experimental Setup and Procedure

Static calibration was conducted for marker-based motion capture system to estimate segment orientation. After 6mn of habituation on the treadmill at a comfortable pace [25], all participants were asked to walk barefoot at three different speeds for 3 min each (slow: 0.7; medium: 1.0; fast: 1.3 m/s). Between each speed, a thirty-second break was given if needed.

To assess the inter-session reliability of the 3D MMC system, participants repeated the same procedure one week later for a second time in the same order.

### 2.3. Data Recording and Processing

#### 2.3.1. Marker-Based System (MOCAP)

**Figure 1:**
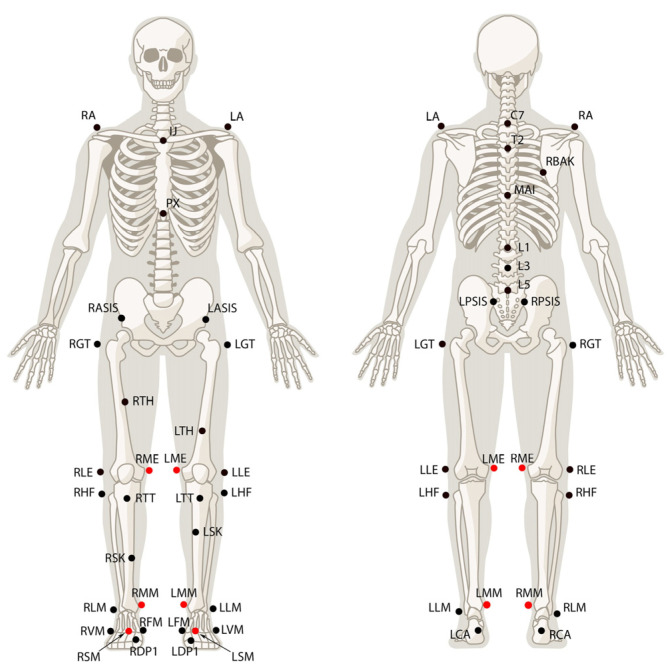
Rizzoli body markerset for OptiTrack (32 Markers).

Height and body mass were recorded before the placement of the markers. Thirty-two passive reflective markers were placed on the anatomical landmarks of the participant according to Rizzoli Body marker set protocol (Figure 1). Prior to the test, participants were asked to wear black shorts and a black shirt. Acquisitions were recorded simultaneously at 100 Hz with a set of 9 infrared OptiTrack Motive 2.2.0 cameras (NaturalPoint, Inc., Corvallis, OR, USA). Two force plates placed under the treadmill were used to measure ground reaction forces at a frequency of 1000 Hz. The gait segmentation was determined from the heel-strike recorded by the force plates. Data recorded by OptiTrack system were exported as C3D files. The markers were used to construct a skeletal model that was scaled with height and mass information, and joint angle kinematics were computed using OpenSim IK tools [26] and Matlab (MATLAB, version R2023a. Natick, MA, USA: The MathWorks Inc. Retrieved from https://www.mathworks.com/) for the right hip and knee joints. In this study, hip and knee joints were given 6 degrees of freedom.

#### 2.3.2. Markerless System (3D MMC)

Two-dimensional digital videos were collected with a set of three ZED 2 cameras (Stereolabs, San Francisco, CA, USA) at a frequency of 30 Hz. The three cameras were placed at the front, back, and right side of the participant. The artificial intelligence tool YOLOv8 [27] was used to estimate the pose of the participants for each of the 2D digital videos, displaying a list of 17 key points. More precisely, the YOLOv8x-pose-p6 pre-trained model was used without any other data training. The “neural depth mode” from Stereolabs’ ZED SDK 4.0 was used to obtain the depth of the 2D key points estimated with YOLOv8. The calibration of extrinsic camera parameters using the ZED360 tool from Stereolabs determined the positions and orientations of the cameras in space. This process enabled the merging of the 3D key points from each camera into a single, unified set of 3D key points.

Finally, joint angles for knee and hip were computed directly from the 3D key points using a simple, straightforward method, assuming a rigid body relationship between the adjacent joints (i.e., knee, hip, and shoulder for the hip angle; knee, ankle, and hip for the knee angle). This method provides a quick and efficient estimation of joint angles but assumes that the estimated key points are accurate representations of the joint centers. The gait segmentation for 3D MMC was determined with the minimum knee flexion angle [28]. To smooth the data, a Butterworth low-pass filter was applied to the raw 3D key point trajectories, with a cutoff frequency of 6 Hz. The entire process, from key point extraction to angle computation, was carried out using Python (Python, version 3.9.13. Wilmington, DE, USA: Python Software Foundation. Retrieved from https://www.python.org/).

### 2.4. Data Analysis and Statistical Calculation

All of the following statistical tests were performed with Python (Python 3.9.13). To evaluate the accuracy of the 3D MMC system, Root Mean Square Error (RMSE) and Lin’s Concordance Correlation Coefficient (LCC) with, respectively, their standard deviation and 95% confidence intervals were computed for the whole waveforms to assess agreement between the two methods. Moreover, from 3D MMC and MOCAP values, the averaged minimum, maximum, and Range of Motion were calculated for flexion/extension of the hip and knee joint angles. A Pearson correlation coefficient (r) was calculated, and a Bland–Altman analysis was performed to assess the correlation and possible bias between both systems. For Pearson correlations, coefficients were interpreted as follows: 0.0–0.30 negligent; 0.30–0.50 low; 0.50–0.70 moderate; 0.70–0.90 high; 0.90–1.00 very high [29]. Lin’s Concordance Correlation Coefficients were interpreted as follows: 0.0–0.90 poor; 0.90–0.95 moderate; 0.95–0.99 substantial; 0.99–1.0 almost perfect [30].

Test–retest reliability was determined using Intraclass Correlation Coefficients (ICC_2,1_), Standard Error of Measurement (SEM), and Minimal Detectable Change (MDC) for the maximum, minimum, and Range of Motion (ROM) values. Furthermore, to assess the reliability of the whole waveform, Root Mean Square Error (RMSE) and Lin’s Concordance Correlation Coefficient (LCC) were computed. For the RMSE, SEM, and MDC values, clinical significance was established for differences over 5° [31]. The ICC_2,1_ values were interpreted as follows: 0.0–0.5 poor; 0.5–0.75 moderate; 0.75–0.9 good; 0.9–1.0 excellent [32].

## 3. Results

### 3.1. Inter-Session Reliability

The 3D MMC system demonstrated a high test–retest reliability for hip and knee joint angles across all speeds. The intraclass correlation coefficients (ICC_2,1_) were consistently above 0.75, except for the minimum knee joint angle at 1.3 m/s (ICC_2,1_ = 0.61), with the maximum hip joint angles showing the highest reliability (ICC_2,1_ > 0.90). All minimal detectable change (MDC) values were below 3°, with the lowest MDC observed for the maximum hip joint angles (MDC < 1.5°) (Table 1).

**Figure 2:**
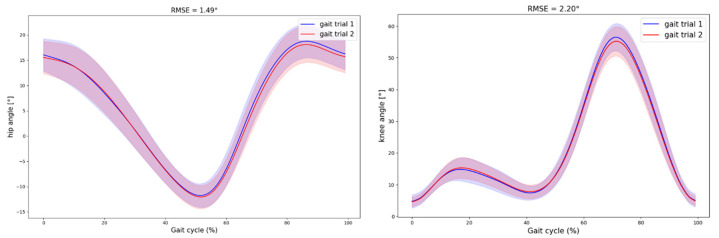
Hip and knee flexion/extension angles waveforms of the 3D Markerless Motion Capture (3D MMC) system at 1.0 m/s. The average gait cycle waveforms with standard deviation of the 15 participants for gait session 1 (blue) and gait session 2 (red) are represented. The associated RMSE values are indicated.

The waveform similarity was also high across the test sessions. The Root Mean Square Error (RMSE) values were all under 2.5°, with the lowest RMSE values observed for the hip joint waveforms (RMSE < 1.80°). Likewise, Lin’s Concordance Correlation Coefficients (LCCs) were ≥0.98, except for the knee joint angles at 0.7 m/s (LCC = 0.96) (Table 1) (Figure 2).

### 3.2. Accuracy Against MOCAP

#### 3.2.1. Non-Corrected Joint Angles

The 3D MMC system showed varying accuracy when compared to MOCAP, with a noticeable constant bias in the joint angle peaks at all speeds. The Bland–Altman plots indicated that most joint angles’ minimum, maximum, and ROM values were within the limits of agreement, though significant constant bias was detected for both knee and hip angles. Pearson’s correlation coefficients for hip angles ranged from 0.13 to 0.53 (except for hip ROM at 0.7 m/s), while for knee angles, Pearson’s r ranged from 0.50 to 0.93 (except for knee maximum angle at 0.7 m/s and 1.0 m/s) (Table 2).

**Figure 3:**
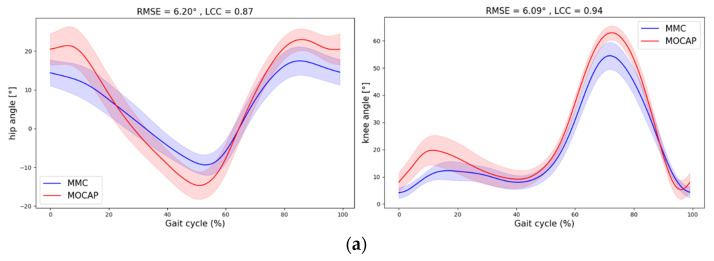

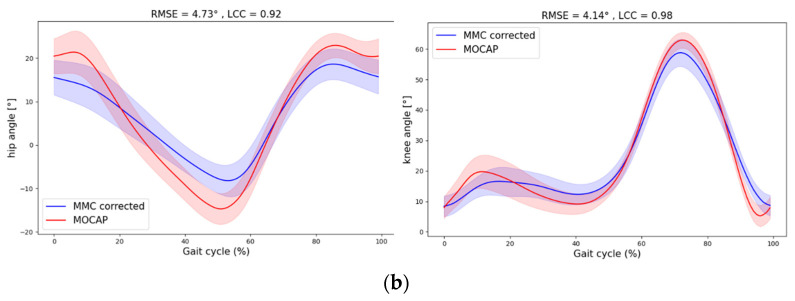
Hip and knee flexion/extension angle waveforms at 1.0 m/s. The average gait cycle waveforms with standard deviation of the 15 participants for 3D MMC (blue) and MOCAP (red) are represented. The associated RMSE and LCC values are indicated. (**a**) Without offset removed; (**b**) offset removed.

The accuracy of the entire waveform showed moderate agreement with MOCAP. The RMSE values for hip and knee joint angle waveforms ranged from 5.86° to 6.80°, while the LCC values were between 0.83 and 0.93 (Table 2) (Figure 3).

#### 3.2.2. Corrected Joint Angles

After correcting for disparities in joint center localization between the two systems, the accuracy significantly improved. Indeed, a disparity in the localization of shoulder, hip, knee, and ankle joint centers between 3D MMC and MOCAP systems resulted in an offset in the kinematic waveform of joint angles. This offset was corrected by subtracting the average difference in the entire waveforms between the MOCAP and 3D MMC systems for each gait session. Following this adjustment, the same statistical analysis was used and the results are presented in Table 3:

**Table 3:** Accuracy of 3D MMC system with offset removed against MOCAP. Bland–Altman bias and Pearson’s r coefficient are presented for maximum, minimum, and ROM values of the hip and knee flexion/extension joint angles. RMSE and LCC values for the entire knee and hip joint flexion/extension angle waveforms are presented.

Joint (Sagittal Plan)	Speed [m/s]	Max		Min		ROM		RMSE [°] (SD)	LCC (95% CI)
		B-A Bias [°] (LoA)	r	B-A Bias [°] (LoA)	r	B-A Bias [°] (LoA)	r		
Knee	0.7	5.04 (−2.47 12.56)	0.58	−2.54 (−5.39 0.30)	0.90	7.59 (−2.05 17.23)	0.50	4.10 (1.58)	0.96 (0.94 0.98)
	1	3.86 (−4.37 12.08)	0.45	−3.43 (−6.37 −0.48)	0.91	7.28 (−2.83 17.39)	0.54	4.14 (1.56)	0.97 (0.95 0.98)
	1.3	2.46 (−3.72 8.63)	0.73	−3.90 (−6.34 −1.47)	0.94	6.36 (−0.95 13.67)	0.65	4.85 (1.33)	0.96 (0.95 0.97)
Hip	0.7	4.23 (2.34 6.12)	0.96	−5.19 (−7.67 −2.72)	0.95	9.42 (5.61 13.23)	0.81	4.14 (0.95)	0.91 (0.88 0.95)
	1	4.49 (2.10 6.89)	0.94	−6.33 (−9.75 −2.92)	0.89	10.82 (5.6 16.05)	0.53	4.73 (0.92)	0.91 (0.89 0.93)
	1.3	5.64 (2.38 8.90)	0.89	−7.03 (−10.73 −3.33)	0.86	12.67 (6.83 18.51)	0.52	5.40 (0.84)	0.91 (0.90 0.93)

ROM (Range of Motion), MMC (Markerless Motion Capture), RMSE (Root Mean Square Error), LCC (Lin’s Concordance Correlation Coefficient), B-A Bias (Bland–Altman Bias), LoA (limit of agreement), SD (standard deviation).

**Figure 4:**
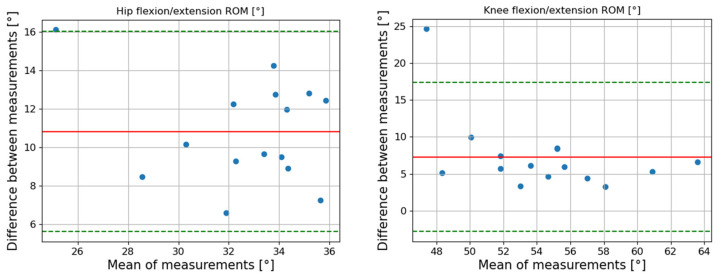
Bland–Altman plots for hip and knee flexion/extension ROM at 1.0 m/s, offset removed. The mean differences are represented (red) along with the 95% limit of agreement (green).

Post-correction, all RMSE values were below 5°, except for the hip joint angle at 5.40° at 1.3 m/s. The LCC values increased to 0.91 for hip angle waveforms and were above 0.96 for the knee angle waveforms (Figure 3). Further improvements were seen in joint angle measurements post-correction. The Bland–Altman plots continued to show constant bias across the maximum, minimum, and ROM measurements of the knee and hip angles (Figure 4). The Pearson’s r coefficients were above 0.81 for all hip joint angle measurements (except for ROM at 1.0 and 1.3 m/s) and above 0.90 for the minimum knee joint angles. The Pearson’s r for the maximum and ROM knee joint angles ranged between 0.50 and 0.73, except for maximum knee joint angle at 1.0 m/s (Table 3).

## 4. Discussion

The aim of this study was to assess the reliability and accuracy of a 3D Markerless Motion Capture (3D MMC) system against a Motion Capture (MOCAP) system. The two hypotheses were validated as the results indicated excellent reliability during test–retest methods and a moderate-to-excellent accuracy compared to MOCAP. However, the results highlighted the presence of constant biases around the maximum, minimum, and ROM values of the hip and knee flexion/extension joint angles.

The 3D MMC system showed excellent test–retest reliability across different gait speeds (slow, medium, and fast), with ICC_2,1_ values above 0.75 for all speeds and conditions, except for the minimum knee joint angle at 1.3 m/s. These finding are consistent with previous research [33]. For example, Balta et al. [33] found that markerless systems using depth cameras like the Kinect could reliably measure gait parameters in the sagittal plane (ICC_2,1_ > 0.85). The high LCC values (>0.96) and low RMSE values (<3°) found in the present study further underscore the 3D MMC system’s reliability, consistent with the findings of Kanko et al. [34], who found an inter-session variability below 3° using multiple digital cameras, indicating clinical precision. These findings indicated that, from one gait test to the other, any measured variation would likely be due to a change in the gait pattern itself, and would not be an error from the system, especially for the hip joint (RMSE and MDC < 2°). Notably, the 3D MMC system’s inter-session reliability may be even higher than the marker-based system’s due to the absence of markers. Indeed, marker placement in traditional MOCAP systems can introduce variability and error up to 5°, as it relies on precise positioning by the operator, which can vary between each session [9]. Therefore, the 3D MMC system might be more advantageous than the traditional MOCAP for test–rest reliability.

The accuracy of the 3D MMC system was then assessed in comparison to MOCAP for each gait speed. Initially, the 3D MMC system exhibited significant discrepancies in joint angle measurements (LCC < 0.90 and RMSE > 7°). These discrepancies could be explained by the differing joint angle localization methods of the two systems, resulting in noticeable offsets between their joint angle measurements (Figure 3). Such biases and offsets have already been reported in previous studies. For instance, Thomas et al. [22] used a single depth camera for gait analysis, and Kanko et al. [16], utilized multiple digital cameras, finding offsets resulting in significant RMSE values, particularly for the hip joint (RMSE of 11°). Two main factors may have contributed to the presence of the offsets found in the present study and in the literature. First, pose estimators are not primarily designed for precise biomechanical analysis, as they are trained on large open access datasets, making them prone to large-scale mislabeling of certain joints, like the hip joint [35]. Second, as Liu and Chang [24] reported in their study combining depth and 2D estimations, the depth estimation in this present study and in Thomas et al.’s [22] only captured the depth of the closest surface to the camera, rather than the precise joint location, leading to differing joint localizations between the two systems. Therefore, to assess the precision of the 3D MMC system without the offset undermining the results, there was a need to remove it. The method used for the offset removal was the same used by Nüesch et al. [36], and more recently by Piche et al. [37]. In these two studies assessing the precision of an inertial measurement unit system against the MOCAP, the offset was removed by subtracting the average difference in the entire waveforms between both systems for each gait session. The results of these studies improved significantly after the offset removal. This reinforces the idea that correcting the signals in the present study might improve the concordance between MOCAP and 3D MMC.

After offset correction of the 3D MMC system for both hip and knee angles, the results significantly improved (Table 3, Figure 3). The 3D MMC system assessed in this study showed a moderate-to-high accuracy compared to MOCAP for the entire waveform at all speeds. Almost all of the RMSE values were below the clinical precision threshold [31], and LCC values showed moderate-to-substantial concordance. These results were in line with the literature, although it is important to note that the studies did not apply similar corrections. For example, Balta et al. [33] recently found their 3D MMC systems using a depth camera to be highly accurate in comparison to MOCAP when the camera was pointed at the side of the individual (RMSE < 4.5°). Similarly, Kanko et al. [16] demonstrated that 3D MMC using multiple digital cameras was an effective method to measure hip or knee joint angle in gait analysis (RMSE < 3.6°). If the 3D MMC system assessed in this present study showed a high accuracy over the entire waveform, only a moderate-to-high accuracy, as well as constant biases, were found for a specific event in the gait cycle (maximum and minimum joint angle and Range of Motion). There are two reasons that can explain these differences. The first reason is that a 30 Hz sample rate was used to record the participants with the 3D MMC system, compared to a 100 Hz sample rate for the MOCAP system. Due to spatial restrictions in the acquisition room, a sample rate of 30 Hz was necessary to capture the wide angle of view of the ZED 2 cameras. While this sample rate may have allowed for a high tracking resolution, it is not surprising that some differences in the peak values were found between the 3D MMC and MOCAP, as Nakano et al. [38] reported previously. They demonstrated that a higher sample rate (120 Hz) provided a greater accuracy than lower sample rates (30 Hz) compared to MOCAP, especially for high-speeds movements. The second reason explaining these contrasts resides in the differences in kinematic computation of joint angles between 3D MMC and MOCAP. In the present study, the angles were computed directly from the 3D key points through direct kinematics. This approach is quick, highly repeatable [39] and thus practical for applications requiring substantial precision in short time, such as longitudinal tracking and training interventions. However, direct kinematics differs fundamentally from the inverse kinematic computations used in MOCAP. This discrepancy arises because direct kinematics does not consider the underlying biomechanical relationships and joint constraints modeled in inverse kinematics. Additionally, for the 3D MMC system, hip flexion/extension was calculated based on a vector constructed from the hip and shoulder key points, and the femur vector. Therefore, beyond not computing the exact hip joint angle, the compensatory movement of the shoulder during gait could have led to an underestimation of true hip flexion and extension, potentially affecting the results. Therefore, the differences in the joint angle measurements between 3D MMC and MOCAP observed in this study can be partly attributed to these methodological distinctions.

In this study, the 3D MMC system showed excellent inter-session reliability, and a good accuracy compared to the MOCAP system, even if constant biases were found. Nevertheless, a few limitations need to be considered when interpreting the findings of this study. First, this study exclusively involved healthy participants, highlighting the need for further research to explore the application of 3D MMC in populations with a pathological gait issue, as it could provide valuable insights [18,40]. Second, although the low-speed gait task used in this study approximated the average gait speed of older adults [41], this does not conclusively demonstrate that this 3D MMC system is effective in older populations. Third, due to spatial constraints in the acquisition room, the cameras were positioned at the front, back and right side of the participants. Therefore, it is not impossible that the 3D MMC system might have produced slightly different results if the cameras were placed in different locations. Fourth, the pose estimation model used in this study only displayed 17 key points on the body, with only one on each foot. Thus, it was not possible to evaluate the ankle joint kinematics. Therefore, an improvement of the 3D MMC system would be to retrain the pre-trained pose estimation model to display an additional key point on each foot to allow the ankle joint angle to be computed.

## 5. Conclusions

This study demonstrates that the 3D MMC system offers excellent reliability and moderate-to-good accuracy compared to the traditional MOCAP system for gait analysis. The high test–retest reliability ensures that the changes observed in gait patterns are due to actual variations in the subject’s movement rather than errors in the system. While the initial assessments revealed some inaccuracies, these were largely attributed to differences in the joint angle localization methods. After correcting these offsets, the accuracy of the 3D MMC system improved significantly, with RMSE values below 5° for hip and knee joint angles. The 3D MMC system’s ability to provide reliable and reasonably accurate measurements without the use of markers presents a significant advantage, especially for long-term patient monitoring and clinical applications where ease of use and patient comfort are priorities. Future improvements, such as integrating a biomechanical model and increasing the sample rate, could further enhance the precision of this system. Overall, the 3D MMC system is a promising tool for gait analysis, offering a practical and reliable alternative to traditional MOCAP systems.

## Figures and Tables

**Table 1:** Reliability of 3D MMC system. ICC_2,1_, SEM, MDC for maximum, minimum, and ROM values of the hip and knee flexion/extension joint angles are presented. RMSE and LCC values for the entire knee and hip joint flexion/extension angle waveforms are presented.

Joint (Sagittal Plan)	Speed [m/s]	Max			Min			ROM			RMSE [°] (SD)	LCC (95% CI)
		ICC_2,1_ (95% CI)	SEM [°]	MDC [°]	ICC_2,1_ (95% CI)	SEM [°]	MDC [°]	ICC_2,1_ (95% CI)	SEM [°]	MDC [°]		
Knee	0.7	0.85 (0.63 0.95)	1.78	2.46	0.85 (0.61 0.95)	0.66	0.92	0.85 (0.63 0.95)	1.85	2.56	2.34 (0.90)	0.96 (0.93 0.99)
	1	0.82 (0.49 0.94)	1.85	2.56	0.86 (0.64 0.95)	0.80	1.11	0.81 (0.48 0.93)	2.20	3.00	2.20 (0.97)	0.99 (0.98 0.99)
	1.3	0.86 (0.59 0.96)	1.60	2.22	0.61 (0.14 0.85)	1.19	1.64	0.82 (0.53 0.93)	2.00	2.77	2.42 (1.44)	0.98 (0.97 1.00)
Hip	0.7	0.90 (0.74 0.97)	1.01	1.40	0.84 (0.57 0.94)	1.37	1.9	0.80 (0.52 0.93)	1.26	1.74	1.80 (0.82)	0.98 (0.98 0.99)
	1	0.94 (0.83 0.98)	0.83	1.15	0.77 (0.44 0.92)	1.22	1.69	0.80 (0.50 0.93)	1.47	2.04	1.49 (0.60)	0.98 (0.97 0.99)
	1.3	0.90 (0.71 0.97)	1.05	1.45	0.78 (0.46 0.92)	1.05	1.45	0.84 (0.59 0.94)	1.02	1.41	1.80 (1.18)	0.98 (0.96 0.99)

ROM (Range of Motion), MMC (Markerless Motion Capture), RMSE (Root Mean Square Error), ICC (intraclass correlation coefficient), SEM (Standard Error of Measurement), MDC (Minimum Detectable Change), CI (confidence interval), SD (standard deviation), LCC (Lin’s Concordance Correlation Coefficient).

**Table 2:** Accuracy of 3D MMC system against MOCAP. Bland–Altman bias and Pearson’s r coefficient are presented for maximum, minimum, and ROM values of the hip and knee flexion/extension joint angles. RMSE and LCC values for the entire knee and hip joint flexion/extension angle waveforms are presented.

Joint (Sagittal Plan)	Speed [m/s]	Max		Min		ROM		RMSE [°] (SD)	LCC (95% CI)
		B-A Bias [°] (LoA)	r	B-A Bias [°] (LoA)	r	B-A Bias [°] (LoA)	r		
Knee	0.7	9.2 (0.3 18.0)	0.41	1.6 (−3.2 6.5)	0.7	7.6 (−2.0 17.2)	0.5	6.00 (2.05)	0.92 (0.88 0.93)
	1	8.2 (−1.6 18.0)	0.46	0.9 (−2.5 4.3)	0.93	7.2 (−2.8 17.4)	0.54	6.09 (2.10)	0.93 (0.90 0.96)
	1.3	7.0 (0.1 13.9)	0.63	0.6 (−3.9 5.2)	0.71	6.4 (−1.0 13.7)	0.65	6.80 (1.60)	0.92 (0.90 0.94)
Hip	0.7	6.1 (−2.0 14.2)	0.14	−3.3 (−12.1 5.4)	0.29	9.4 (5.6 13.2)	0.81	5.86 (1.70)	0.83 (0.80 0.87)
	1	5.7 (−2.8 14.2)	0.19	−5.1 (−13.4 3.1)	0.13	10.8 (5.6 16.0)	0.53	6.20 (1.50)	0.86 (0.82 0.89)
	1.3	6.9 (−1.5 15.4)	0.28	−5.7 (−13.7 2.2)	0.14	12.7 (6.8 18.5)	0.52	6.62 (1.60)	0.88 (0.85 0.90)

ROM (Range of Motion), MMC (Markerless Motion Capture), RMSE (Root Mean Square Error), LCC (Lin’s Concordance Correlation Coefficient), B-A Bias (Bland–Altman Bias), LoA (limit of agreement), SD (standard deviation).

## Data Availability

The raw data and descriptive statistics are contained within the Supplementary Materials. The camera recordings (.C3D and ZED2 cameras recordings) are available on request from the corresponding author.

## Supplementary Materials

The following supporting information can be downloaded at: https://www.mdpi.com/article/10.3390/s24227105/s1; Table S1: dataset.xlsx.
